# Vision difficulty and dementia: economic hardships among older adults and their caregivers

**DOI:** 10.3389/fepid.2023.1210204

**Published:** 2023-08-03

**Authors:** Priyanka Kumar, Grace Chung, Emmanuel Garcia-Morales, Nicholas S. Reed, Orla C. Sheehan, Joshua R. Ehrlich, Bonnielin K. Swenor, Varshini Varadaraj

**Affiliations:** ^1^ Department of Medicine, University of Maryland Medical Center Midtown Campus, Baltimore, MD, United States; ^2^ Department of Health Management and Policy, University of Michigan School of Public Health, Ann Arbor, MI, United States; ^3^ Cochlear Center for Hearing and Public Health, Johns Hopkins Bloomberg School of Public Health, Baltimore, MD, United States; ^4^ Johns Hopkins Disability Health Research Center, Johns Hopkins University, Baltimore, MD, United States; ^5^ Department of Epidemiology, Johns Hopkins Bloomberg School of Public Health, Baltimore, MD, United States; ^6^ The Johns Hopkins Center on Aging and Health, Johns Hopkins University, Baltimore, MD, United States; ^7^ RCSI Hospital Group, Connolly Hospital, Dublin, Ireland; ^8^ Department of Ophthalmology and Visual Sciences, University of Michigan, Ann Arbor, MI, United States; ^9^ Institute for Social Research, University of Michigan, Ann Arbor, MI, United States; ^10^ The Johns University School of Nursing, Johns Hopkins University, Baltimore, MD, United States

**Keywords:** vision difficulty, dementia, economic hardships, older adults, caregiver financial difficulty

## Abstract

**Introduction:**

Limited research has examined the economic impact of vision difficulty (VD) and dementia on older adults and their caregivers. We aimed to determine whether older adults with VD and/or dementia, and their caregivers, face more economic hardships than their counterparts without VD or dementia.

**Methods:**

We used cross-sectional data from the 2015 National Health and Aging Trends Study (NHATS), a population-based survey of Medicare beneficiaries, linked to their family/unpaid caregivers from the National Study of Caregiving (NSOC). Regression models characterized the association of VD (self-report), dementia (survey and cognitive assessments), and co-occurring VD and dementia with debt, receiving financial help from relatives, government-based Supplemental Nutrition Assistance Program (SNAP), other food assistance, utility assistance, and caregiver financial difficulty.

**Results:**

The NHATS sample included 6,879 community-dwelling older adults (5670 no VD/dementia, 494 VD-alone, 512 dementia-alone, 203 co-occurring VD and dementia). Adults with VD and dementia had higher odds of receiving SNAP benefits (OR = 2.6, 95%CI = 1.4–4.8) and other food assistance (OR = 4.1, 95%CI = 1.8–9.1) than adults without VD/dementia, while no differences were noted for debt, financial help, and utility assistance. Adults with VD-alone had higher odds of debt (OR = 2.1, 95%CI = 1.3–3.2), receiving financial help (OR = 1.7, 95%CI = 1.1–2.5) and other food assistance (OR = 2.7, 95%CI = 1.7–4.3); while adults with dementia-alone had higher odds of debt (OR = 2.8, 95%CI = 1.4–5.5). The NSOC sample included 1,759 caregivers (995 caring for adults without VD/dementia, 223 for VD-alone, 368 for dementia-alone, and 173 for co-occurring VD and dementia). Compared to caregivers of older adults without VD/dementia, caregivers of adults with VD and dementia had higher odds of financial difficulty (OR = 3.0, 95%CI = 1.7–5.3) while caregivers of adults with VD-alone or dementia-alone did not.

**Discussion:**

While older adults with VD- or dementia-alone experienced increased economic hardships, disparities in food assistance were amplified among older adults with co-occurring disease. Caregivers of adults with co-occurring disease experienced more financial difficulty than caregivers of adults with a single or no disease. This study highlights the need for interventions across clinical and social services to support the economic wellbeing of our aging population and their caregivers.

## Introduction

As the global population ages, it is becoming increasingly important to develop strategies to support the health and well-being of older adults and their caregivers (1, 2). Aging populations carry with them an increased prevalence of chronic disease, including vision difficulty (VD) and dementia (3, 4). Older adults with co-occurring sensory and cognitive difficulties are at increased risk for magnified caregiving needs and healthcare expenditures (5, 6). These financial costs are substantial; the Alzheimer’s Association estimated the costs of unpaid dementia caregiving at over $270 billion in 2021 (7).

Economic security is central not only to older adults’ independence and well-being but also their caregivers’ welfare and capacity for caregiving (8, 9). Research on the financial capacity of older adults with dementia revealed challenges in domains ranging from monetary skills to conceptual knowledge, to financial judgement, all exacerbated with increasing disease severity (10). However, despite existing evidence establishing the relationship between vision and cognition, the role of co-occurring dementia and VD on financial implication for older adults and their caregivers is an overlooked aspect. While the concurrence of any two or more aging health conditions may be postulated to impact financial hardships, data show that co-occurring vision and cognitive impairments have a particularly strong and distinct impact on functioning (11, 12). Further, vision loss has been established as a risk factor for cognitive decline and dementia (13, 14). Therefore, understanding the interplay between vision and cognitive impairment and their cumulative impact on financial hardships is essential to plan for appropriate supportive interventions.

Diminished financial skills among older adults have also been identified as the strongest predictor of time-burden and hostility among caregivers (15). In previous research linking U.S. national samples of older adults and their caregivers, caring for older adults with VD was found to involve similar time demands as caring for older adults with dementia, but impacts on time demands and participation in valued activities were greater when caring for older adults with both dementia and VD (5). Caregivers face indirect costs of care (e.g., hours of informal care, opportunity cost of missed employment) in addition to direct costs (e.g., medications, consultations) (16). Consequently, a better understanding of economic outcomes among caregivers to older adults with chronic disease is also critical to identify areas to target financial resources and interventions.

Here, using nationally representative data, we build on prior work to examine economic outcomes among older adults with VD and dementia linked to their caregivers across various domains, ranging from debt to food security and assistance. We hypothesized that VD and dementia not only pose substantial economic strains on older adults and their caregivers, but also that this strain is amplified when VD and dementia co-occur.

## Materials and methods

### Study population

This study population comprised of data from the 2015 National Health and Aging Trends Study (NHATS) linked to the 2015 National Study of Caregiving (NSOC). This linked 2015 NHATS-NSOC dataset provides a cross-sectional perspective of the habits and wellbeing of older adults and their caregivers in the United States. This secondary analysis of publicly available data was acknowledged as exempt research by the institutional review board of the Johns Hopkins School of Medicine.

NHATS draws on annual in-person interviews with a nationally representative sample of Medicare beneficiaries aged ≥65 (17). Periodically, family and unpaid helpers of the NHATS older adults who received help with self-care, transportation, mobility, household activities, and medical tasks are eligible for participation in NSOC. Up to five helpers for each NHATS participant are interviewed in NSOC (18).

#### Analytic sample

**Figure 1:**
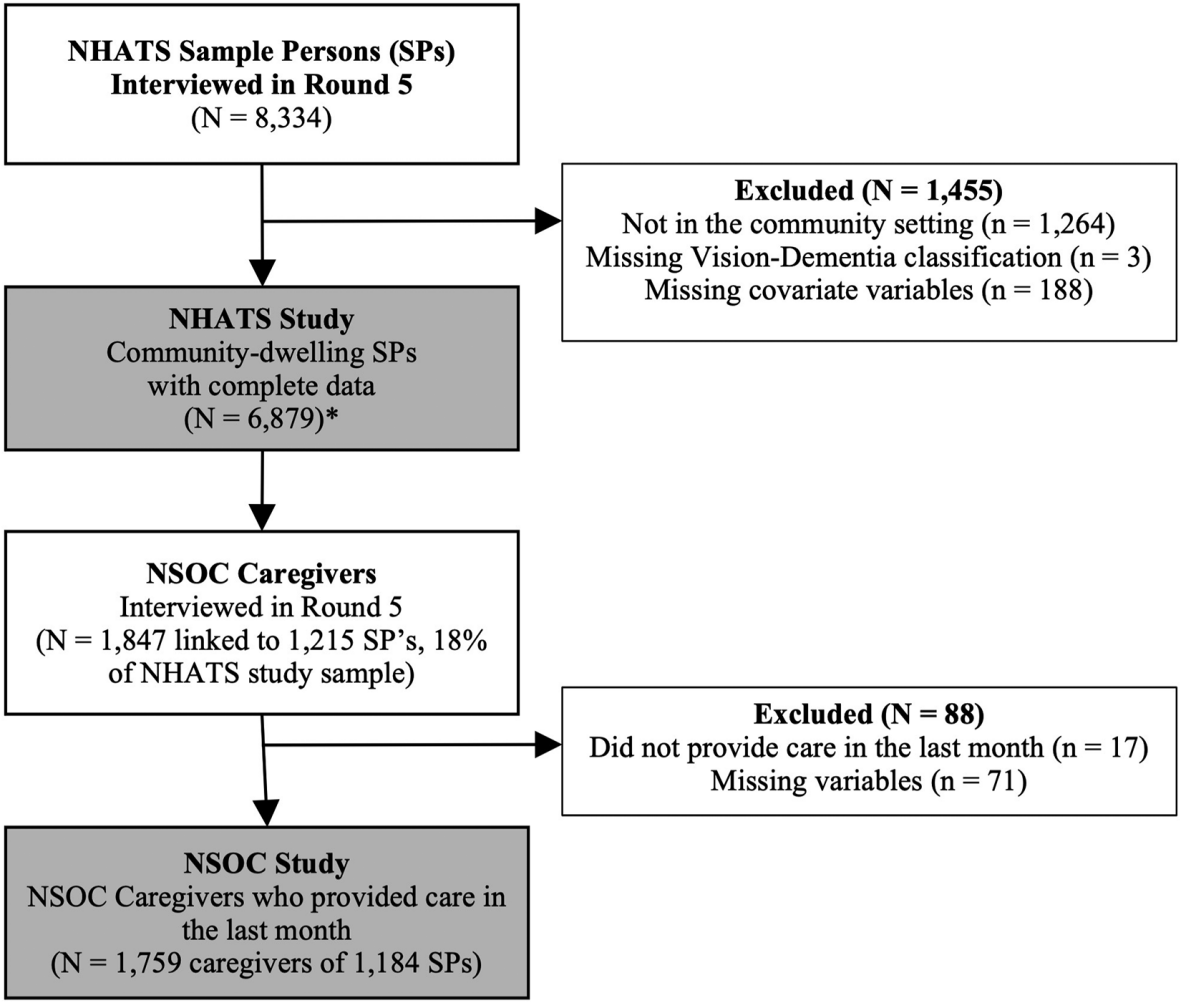
Study participation selection, NHATS and NSOC 2015.

This analytic sample included all community dwelling NHATS adults from the 2015 cohort (*n* = 6,879) with either complete data for all study variables or imputed values provided by NHATS when response data were not available (Figure 1). Older adults who were not community dwelling (i.e., residing in residential care facilities) were excluded from this analysis (*n* = 1,264). We limited this study to all NSOC caregivers who had assisted an NHATS participant with any activity in the last month. The final caregiver analytic sample consisted of 1,759 caregivers linked to 1,184 NHATS older adults (Figure 1).

### VD/dementia status among older adults

Based on a classification scheme for cognitive function devised by NHATS and self-reported visual disability, a dual VD and probable dementia status variable was generated (19). The variable was binned as one of four categories, as previously done (5): (1) no VD or probable dementia; (2) VD alone; (3) probable dementia alone; (4) VD and probable dementia.

#### Vision difficulty

VD was defined by participant- or proxy-reported difficulty with distance or near vision while using contact lenses or glasses, as previously done (5, 20). Specifically, VD was defined as not seeing well enough in any of the following vision domains: (1) recognizing someone across the street, (2) watching television across the room, and (3) reading newspaper print.

#### Cognitive impairment

Cognitive difficulty status was categorized based on survey report, response to a validated dementia questionnaire, and performance on cognitive tests (19). Difficulty was classified into three groups: probable dementia, possible dementia, or no dementia. Survey report of dementia status, provided by either the NHATS older adult or proxy respondent, was defined as having a physician diagnosis of dementia or Alzheimer disease. For proxy interviews, the Eight-item Informant Interview to Differentiate Aging and Dementia (AD8), a valid and reliable screening tool to detect early cognitive changes, was administered (21). Cognitive testing evaluated memory, orientation, and executive function. Criteria for probable dementia classification included: diagnosis of dementia, scoring ≥2 on the AD8, and falling ≤1.5 standard deviations (SD) below the mean in at least two domains on cognitive testing. Possible dementia classification included falling ≤1.5 SD below the mean in one domain on cognitive testing. Study participants who did not meet criteria in any domain were classified as having no dementia. Though our primary analysis considered only individuals with probable dementia, further sensitivity analysis combined the subgroups of probable and possible dementia to assess a broader and more inclusive definition of dementia status (Supplementary Data).

### Economic outcome measures

#### NHATS older adults

Among NHATS adults, economic outcomes (all binary variables) included: credit card debt, receiving financial help, Supplemental Nutrition Assistance Program (SNAP) assistance, other food assistance (e.g., Meals-on-Wheels), and financial assistance with utilities. A participant was defined as having credit card debt if they reported only paying the minimum amount due in response to “Do you usually pay off all credit card balances every month or only the minimum amount due?”. A participant was defined as receiving financial help if they responded yes to “Last year, did you receive any financial help or gifts from relatives, either regularly—like every month—or just every so often as needed?”. Indicator variables were constructed based on participant response to “There are several state and federal programs that help people in need. In the last year, did you receive help from any of these programs?” for SNAP, other food assistance such as Meals-on-Wheels, or receipt of gas, electricity, or other energy assistance.

#### NSOC caregivers

Financial difficulty for NSOC caregivers was defined based on the response to “Helping older relatives or a spouse or partner who has health problems can be difficult. Is helping them financially difficult for you?”.

### Other variables

Demographic variables among NHATS participants included age (continuous), gender (male/female), race/ethnicity (Non-Hispanic White/Black/other, Hispanic), education (high school or less, some college/vocational, completed college), marital status (married/living with partner, separated/divorced/widowed/never married), number of children (0, 1, >1), number of comorbidities (0–1, 2–3, 4+), and hearing difficulty (no/yes). Comorbid conditions included diabetes, hypertension, arthritis, osteoporosis, lung disease, stroke, heart disease, cancer, and hip fracture. NSOC demographic variables included age (continuous), gender (male/female), education (high school or less, some college/vocational, completed college), self-rated health (“high” capturing excellent/very good/good, and “low” capturing fair/poor), and relationship to the older adult (spouse, daughter/son, other relative, nonrelative).

### Statistical analysis

Differences in older adult and caregiver characteristics by VD and probable dementia status were assessed using Pearson chi-squared test for categorical variables and one-way analysis of variance for continuous variables. All analyses accounted for the multistage complex survey design of NHATS and NSOC using study-provided weights.

We conducted multivariable logistic regression analyses to examine economic difficulties by VD and probable dementia status for NHATS participants and their NSOC caregivers, accounting for clustering by care recipient. Propensity score (PS) weighting (using multinomial regression, and including age, gender, race, education, and marital status) was used to account for differences among the four NHATS groups. To get a good non-linear fit of age, we used natural spline transformation of age.

The models for NHATS older adults were adjusted for age, gender, race, education and marital status, comorbidity score, hearing difficulty, and the number of children. These older adult characteristics as well as caregiver age, sex, education, self-reported health, and relationship to the recipient were included as covariates in the models for NSOC caregivers. The final survey weights in the regression models included the use of inverse PS weights trimmed to the 96th percentile computed for the average treatment effect (ATE) and multiplied by the NHATS final analytic weights. Two-sided *p* values < .05 were considered statistically significant. All analyses were performed using R version 4.1.3 and Stata version 17.0.

## Results

### Characteristics of NHATS older adults

The NHATS analytic sample included 6,879 older adults, of whom 5,670 (87.2%) reported no VD or dementia, 494 (6.2%) reported VD alone, 512 (4.8%) reported dementia alone, and 203 (1.8%) reported both VD and dementia (Table 1A). The mean age of NHATS participants was 74.5 years (SE = 7.0), the majority identified as female (57.2%), non-Hispanic White (66.6%), were married/living with a partner (53.0%), had 0–1 comorbid conditions (61.0%), and did not have hearing difficulty (75.6%) (Table 1A). There were sociodemographic differences between the four groups as detailed in Table 1A.

**Table 1A:** Participant characteristics by VD and dementia status, NHATS 2015.

Demographic characteristic	Total *N* = 6879	No VD or dementia *N* = 5670 (87.2%)	VD alone *N* = 494 (6.2%)	Dementia alone *N* = 512 (4.8%)	VD and dementia *N* = 203 (1.8%)	*p*-value
Age, mean (SE)	74.5 (7.0)	73.9 (6.6)	75.7 (8.0)	81.1 (7.6)	81.3 (8.3)	<0.001
Age, in years, *n* (%)						
65–69	996 (14.5)	910 (32.2)	66 (29.2)	13 (7.2)	7 (9.4)	<0.001
70–74	1677 (24.3)	1511 (28.6)	95 (22.7)	53 (14.8)	18 (15.9)	
75–79	1501 (21.8)	1291 (19.1)	109 (19.6)	77 (18.8)	24 (17.3)	
80–84	1277 (18.6)	1017 (11.5)	83 (11.5)	130 (23.6)	47 (20.8)	
85–89	890 (13.0)	630 (6.1)	84 (11.2)	127 (22.2)	49 (18.3)	
90+	538 (7.8)	311 (2.5)	57 (5.9)	112 (13.5)	58 (18.3)	
Female, *n* (%)	3933 (57.2)	3188 (54.1)	325 (64.2)	294 (51.3)	126 (56.8)	
Race/Ethnicity, *n* (%)						
Non-Hispanic white	4758 (66.6)	4100 (82.1)	283 (67.9)	284 (68.2)	91 (56.7)	<0.001
Non-Hispanic black	1481 (21.5)	1120 (7.9)	136 (11.5)	153 (12.6)	72 (15.3)	
Non-Hispanic Other	210 (3.1)	163 (3.8)	19 (4.5)	20 (4.6)	8 (7.4)	
Hispanic	430 (6.3)	287 (6.2)	56 (16.1)	55 (14.6)	32 (20.5)	
Education, *n* (%)						
≤High school	3370 (49.0)	2534 (39.5)	320 (62.8)	362 (70.5)	154 (75.1)	<0.001
Some college/Vocational	1470 (21.4)	1291 (24.2)	89 (18.4)	66 (11.7)	24 (13.8)	
≥College	2039 (29.6)	1845 (36.3)	85 (18.8)	84 (17.8)	25 (11.1)	
Marital Status, *n* (%)						
Married/living with partner	3641 (53.0)	3151 (61.8)	205 (49.2)	210 (48.9)	75 (44.6)	<0.001
Separated/Divorced/Widowed/Never married	3238 (47.1)	2519 (38.2)	289 (50.8)	302 (51.1)	128 (55.4)	
Number of Children, *n* (%)						
0	564 (8.2)	472 (8.9)	37 (7.1)	42 (7.0)	13 (6.2)	0.499
1	861 (12.5)	690 (12.2)	69 (12.9)	70 (15.1)	32 (14.5)	
>1	5454 (79.3)	4508 (78.9)	388 (80.0)	400 (77.9)	158 (79.4)	
Number of comorbid conditions^a^, *n* (%)						
0–1	4191 (61.0)	3470 (51.9)	280 (43.9)	321 (53.3)	120 (47.6)	<0.001
2–3	1883 (27.3)	1586 (35.3)	124 (31.8)	123 (29.9)	50 (28.4)	
4+	805 (11.7)	614 (12.8)	90 (24.3)	68 (16.8)	33 (24.0)	
Hearing Impairment, *n* (%)						
No	5203 (75.6)	4416 (80.1)	334 (69.8)	347 (64.3)	106 (49.5)	<0.001
Yes	1676 (24.4)	1254 (19.9)	160 (30.2)	165 (35.7)	97 (50.5)	

VD, vision difficulty; NHATS, national health and aging trends study; SE, standard error.

*N* is unweighted; % is survey weighted estimates.

^a^ Comorbid conditions include: diabetes, hypertension, arthritis, osteoporosis, lung disease, stroke, heart disease, cancer, and hip fracture.

### Characteristics of NSOC caregivers

The NSOC population included responses from 1,759 caregivers, of whom 995 (61%) cared for adults without VD or dementia, 223 (14%) for VD alone, 368 (16%) for dementia alone, and 173 (8%) for both VD and dementia (Table 1B). The mean age of NSOC caregivers was 58.1 (SE = 15.8) years, the majority identified as female (66.6%), had high perceptions of self-rated health (78.5%), and identified as offspring of the older adult (54.4%) (Table 1B). Caregivers in the 4 groups largely shared similar demographic characteristics, as detailed in Table 1B.

**Table 1B:** Characteristics of caregivers by NHATS older adult VD and dementia status, NSOC 2015.

Caregiver Characteristics	Total *N* = 1759	No VD or dementia *N* = 995 (61%)	VD alone *N* = 223 (14%)	Dementia alone *N* = 368 (16%)	VD & dementia *N* = 173 (8%)	*p*-value
Age, mean (SE)	58.1 (15.8)	59.0 (15.8)	56.6 (15.8)	58.4 (15.0)	52.6 (16.2)	0.177
Female, *n* (%)	1,172 (66.6)	647 (60.2)	143 (59.3)	264 (70.7)	118 (55.1)	
Education, *n* (%)						
≤High school	705 (40.1)	394 (39.0)	85 (36.3)	152 (43.4)	74 (48.4)	0.082
Some college	621 (35.3)	346 (35.9)	97 (46.8)	119 (33.9)	59 (27.6)	
≥College	433 (24.6)	255 (25.0)	41 (17.0)	97 (22.7)	40 (24.0)	
Self-rated health status, *n* (%)						
High	1,381 (78.5)	783 (78.3)	175 (75.3)	292 (77.1)	131 (74.0)	0.701
Low	378 (21.5)	212 (21.7)	48 (24.7)	76 (22.9)	42 (26.0)	
Relationship to the older adult, *n* (%)						
Spouse	416 (23.6)	277 (28.9)	46 (22.3)	68 (18.5)	25 (12.4)	0.016
Daughter or son	957 (54.4)	489 (44.6)	120 (52.2)	235 (60.4)	113 (67.7)	
Other relative	272 (15.5)	157 (17.3)	34 (13.6)	53 (15.7)	28 (13.8)	
Nonrelative	114 (6.5)	72 (9.1)	23 (11.9)	12 (5.4)	7 (6.1)	

VD, vision difficulty; NHATS, national health and aging trends study; NSOC, national study of caregiving; SE, standard error.

Survey weighted estimates shown.

### Economic outcomes among NHATS older adults

**Figure 2:**
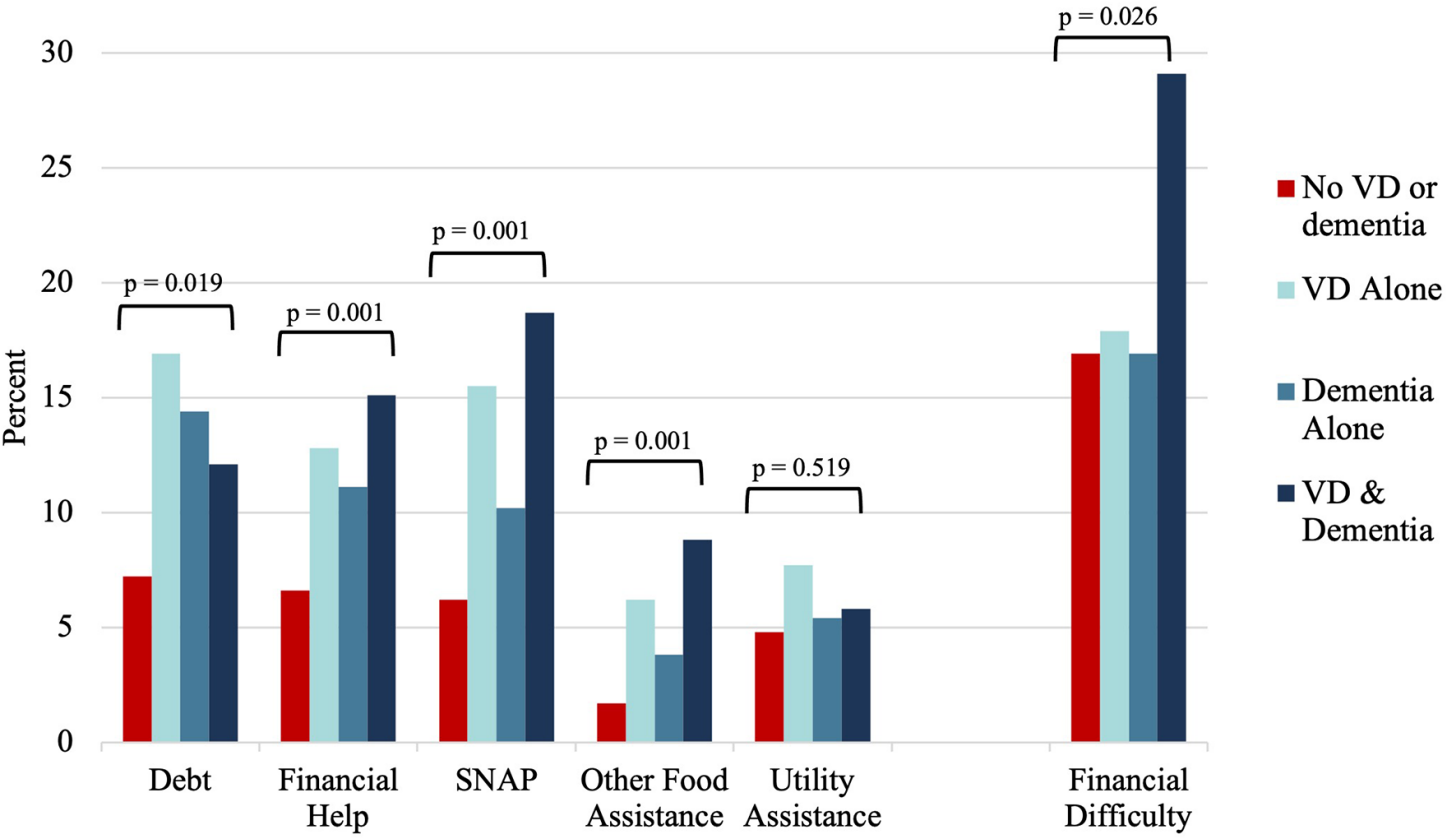
Distribution of unadjusted economic outcomes by vision difficulty and dementia status, NHATS and NSOC 2015. VD, vision difficulty; NHATS, national health and aging trends study; NSOC, national study of caregiving.

In unadjusted analysis, compared to adults without VD or dementia, the other groups faced more debt, and required more financial help, SNAP, other food assistance, and utility assistance (Figure 2).

In fully adjusted logistic regression analyses, compared to older adults without VD or dementia, older adults with VD and dementia had 2.6-fold greater odds of receiving SNAP benefits (95% CI, 1.4–4.8) and 4.1-fold greater odds of utilizing other food assistance programs (95% CI, 1.8–9.1), but there were no differences in the odds of debt (OR = 1.4, 95% 0.4–5.0) or receiving financial help (OR = 1.5, 95% CI 0.8–2.7) (Table 2A).

**Table 2A:** Multivariable regression analyses: economic outcomes by VD status and dementia status, NHATS 2015.

	Model 1: Debt^a^ (*N* = 5,246) OR (95% CI)	Model 2: Financial help^b^ (*N* = 6,754) OR (95% CI)	Model 3: SNAP^c,d^ (*N* = 6,806) OR (95% CI)	Model 4: Other food assistance^c,e^ (*N* = 6,809) OR (95% CI)	Model 5: Utility assistance^c.f^ (*N* = 6,802) OR (95% CI)
No VD or dementia	Reference	Reference	Reference	Reference	Reference
VD alone	2.1 (1.3, 3.2)	1.7 (1.1, 2.5)	1.8 (1.1, 2.9)	2.7 (1.7, 4.3)	1.2 (0.8, 1.8)
Dementia alone	2.8 (1.4, 5.5)	1.4 (0.9, 2.2)	1.6 (0.9, 2.8)	1.6 (0.8, 3.4)	0.8 (0.5, 1.4)
VD & dementia	1.4 (0.4, 5)	1.5 (0.8, 2.7)	2.6 (1.4, 4.8)	4.1 (1.8, 9.1)	0.9 (0.4, 2.2)

VD, vision difficulty; NHATS, national health and aging trends study; SNAP, supplemental nutrition assistance program; OR, odds ratio; CI, confidence interval.

Models adjusted for NHATS participants’ age, sex, race/ethnicity, education, marital status, number of children, comorbidities, and hearing impairment.

^a^ Only pay the minimum amount due in response to- “Do you usually pay off all credit card balances every month or only the minimum amount due?”.

^b^ “Last year, did you receive any financial help or gifts from relatives, either regularly—like every month—or just every so often as needed?”.

^c^ “There are several state and federal programs that help people in need. In the last year, did you receive help from any of these programs?”.

^d^ Food stamps (also called the Supplemental Nutrition Assistance Program, or SNAP).

^e^ Other food assistance such as Meals-on-Wheels?.

^f^ Gas, electricity, or other energy assistance?.

Older adults with VD alone faced higher odds of debt (OR = 2.1, 95% CI, 1.3–3.2), receiving more financial help (OR = 1.7, 95% CI, 1.1–2.5), SNAP benefits (OR 1.8, 95% CI, 1.1–2.9), and other food assistance (OR = 2.7, 95% CI, 1.7–4.3) (Table 2A). Similarly, older adults with dementia alone had higher odds of debt (OR = 2.8, 95% CI, 1.4–5.5), but there were no differences in the odds of receiving financial help (OR = 1.4, 95% CI, 0.8–2.5), SNAP benefits (OR = 1.3, 95% CI, 0.7–2.3) or other food assistance (OR = 1.1, 95% CI, 0.5–2.4) as compared to older adults without VD or dementia (Table 2A). There were no significant differences across older adults by VD/dementia status for utility assistance.

### Economic outcomes among NSOC caregivers

In unadjusted analysis, there were significant group differences in caregiver-reported financial difficulty (Figure 2). In fully adjusted logistic regression analyses, compared to caregivers of older adults with neither difficulty, caregivers of older adults with co-occurring difficulty faced 3.0-fold greater odds of financial difficulty (95% CI, 1.7–5.3), while caregivers of adults with either VD alone (OR = 1.2, 95% CI 0.7–2.2) or dementia alone (OR = 1.0, 95% CI 0.5–1.8) showed no significant differences (Table 2B).

**Table 2B:** Multivariable regression analyses: economic outcomes for caregivers of NHATS older adults by VD and dementia status, accounting for clustering by care recipient, NSOC 2015.

	Model 1: Financial difficulty^a^ (*N* = 1,759) OR (95% CI)
No VD or dementia	*Reference*
VD alone	1.2 (0.7, 2.2)
Dementia alone	1.0 (0.5, 1.8)
VD and dementia	3.0 (1.7, 5.3)

VD, vision difficulty; NHATS, national health and aging trends study; NSOC, national study of caregiving; OR, odds ratio; CI, confidence interval.

Models adjusted for NHATS participants’ age, sex, race/ethnicity, education, marital status, number of children, comorbidities, and hearing impairment, and NSOC caregiver age, caregiver sex, caregiver education, caregiver self-reported health, and caregiver relationship to the older adult.

^a^ Caregivers were asked if helping the NHATS participant was financially difficult for them.

### Sensitivity analysis

In sensitivity analyses examining the broader classification of dementia to include probable and possible dementia, we found similar results (Supplementary Data).

## Discussion

In a nationally representative sample of older adults and their caregivers, older adults with VD and dementia were more likely to receive SNAP benefits and other food assistance as compared to their non-impaired peers. These differences may be attributed to several factors, including: unrealized lifetime income/wealth potential (possibly secondary to employment disparities), greater health care expenditures with increasing disease burden, compounded challenges in accessing care (e.g., resources that are not adaptable for low-vision), and resource limitations unique to food security. These results also found that caregivers of older adults with both VD and dementia experience significantly more financial difficulty than caregivers of adults with VD alone, dementia alone, and neither difficulty. These results support the hypothesis that co-occurring VD and dementia not only creates substantial economic strains on older adults and their caregivers, but also that this strain is amplified with increasing chronic disease burden.

Both VD and cognitive difficulty are independently associated with worse economic outcomes. VD is associated with un- and under-employment, lower income levels, lack of private health insurance, and food insecurity (22–24). Similarly, cognitive difficulty is associated with lower household income, higher medical expenditures, and food insecurity (25, 26). There may be unaddressed vulnerabilities that shape these outcomes among older adults; research has shown that adults face adverse financial events years before the clinical diagnosis of age-based dementia, suggesting a need for the primary prevention of negative economic outcomes (27). Future studies investigating the role of VD in this early decline of financial wellbeing is necessary to identify interventions to support older adults with co-occurring VD/dementia.

While increasing disease burden itself likely presents worse economic outcomes among older adults, the case of dementia and VD has unique associations. First, VD and cognitive difficulty share common risk factors, including inflammation and vascular changes (28). Second, VD has itself been found to be a risk factor for cognitive decline and dementia (13, 14, 29). Notable confounding variables may include the physical decline and worse psychological functioning associated with VD which themselves likely contribute to cognitive difficulty (12, 28).

This work highlights disparities by VD and dementia status of older adults with respect to food security, and more specifically, SNAP benefits and other food assistance programs (e.g., Meals-on-Wheels). Our findings are consistent with data showing that disabilities are associated with food insecurity (30). Notably, low-income adults in the US with VD experience more than twice the odds (OR = 2.16, 95% CI, 2.01–2.31) of being food insecure than their peers without VD, and there is a significant and bi-directional relationship between VD and food insecurity (24, 31). Further, cognitive limitations such as trouble managing money is significantly associated with food insecurity (32).

Our finding of significantly increased financial difficulty among caregivers of adults with dual cognitive and sensory difficulty compared to caregivers of adults with either a single or no difficulty is in line with prior literature showing magnifying caregiving needs at the intersection of these co-occurring impairments (5). Notably, one of the strongest predictors of caregiving burden is the financial expense of caregiving (33, 34). Caregivers of older adults with dementia face significant financial costs, even with external support (i.e., nursing home care); among older adults with dementia, research has demonstrated that family caregivers shoulder over 40% of total expenditures across the last 7 years of life, totaling $60,320 informal caregiving costs and $105,590 out-of-pocket costs (35). These figures amount to significant personal investments on the part of caregivers, who often already face the multi-faceted strains of declining physical health, emotional and psychological stress, strained familial relationships, and social isolation (33).

Although the relationship between food security and chronic health difficulties may be bidirectional, this preliminary and cross-sectional work lends support to the hypothesis that chronic disease may drive food insecurity. To mitigate disparities in food security in an aging population, older adults with cognitive and sensory difficulties may benefit from increased screening for food security. Further, existing food assistance programs can be further optimized to support this population; SNAP has been shown to vary widely state-by-state in accessibility (i.e., low contrast content, animation and movement) and flexibility of enrollment (36). For adults with sensory difficulty, cognitive difficulty, or both, deficiencies in any of these factors create significant hurdles to accessing and navigating this service. Lastly, addressing stigma surrounding food assistance programs like SNAP will enable such programs to better serve their target populations (37).

As the global population ages and the incidence of chronic disease increases, there are significant economic implications. This work highlights key areas for intervention to alleviate pressures faced by older adults, their caregivers, and the health system at large. To support older adults, an emphasis should be made on discussions in the clinic space regarding strategies to support financial wellbeing (e.g., personal account checking) with references made to relevant national organizations (e.g., National Council on Aging’s Benefits Check Up, AARP’s Money Management Program) (8, 38). To maximize the potential of these resources, investments need to be made to ensure that these platforms are accessible for adults with disability. Of note, as VD has been shown to impact financial wellbeing among older adults with dementia, perhaps a broader focus on the role of VD in geriatrics is needed with integration of vision rehabilitation services into geriatric care. Finally, support for caregivers may take the shape of encouraging individual wellbeing (i.e., counseling, support groups, training, respite care) as well as providing resources to national organizations (e.g., National Family Caregiver Support Program).

There are limitations to this work. First, these are cross-sectional data; to examine the temporal link between VD and dementia status with economic outcomes, longitudinal analyses are required. As both dementia and VD represent progressive chronic disease, the relationships between disease status and economic outcomes will likely become stronger with time. Second, NHATS assesses self- and proxy-reported exposure (VD and dementia) variables and financial outcomes, which are susceptible to recall biases. While self-reported VD may measure a different construct than objective vision assessments, research has demonstrated its value in obtaining individual perspectives on function and disability (20, 39). Finally, it can be argued that the participants’ report of VD (or financial outcomes) may be affected by their cognitive status and vice versa; however, self-reported data on VD has been widely used in prior studies examining cognitive outcomes (12).

This study provides a national view of economic disparities by VD and dementia status of older adults and their caregivers. While older adults with either VD or dementia experienced increased economic strains across financial help and food assistance/insecurity, these disparities were amplified among older adults with co-occurring VD and dementia. Caregivers of adults with these co-occurring VD and dementia also experienced more financial difficulty than caregivers of adults with a single or no difficulty. This study highlights areas for intervention across clinical spaces, social services, and national organizations to support the economic wellbeing of our aging population and their caregivers.

## Data Availability

Publicly available datasets were analyzed in this study. This data can be found here: https://www.nhats.org/researcher/nhats.
