# Supplementary material for: Vision difficulty and dementia: economic hardships among older adults and their caregivers
Source: Front Epidemiol. 2023 Aug 3;3:1210204. doi: 10.3389/fepid.2023.1210204 (PMC10910956; doi:10.3389/fepid.2023.1210204)
Supplement: Supplementary file 1 [file Table1.docx]

**Supplemental Data: Sensitivity Analyses**

| **Supplementary Table 1:** Multivariable Regression Analyses: Economic outcomes by VD and probable/possible dementia status, NHATS 2015 | | | | | |
| --- | --- | --- | --- | --- | --- |
| Group | ***Model 1:***  ***Debt^a^***  *OR (95% CI)* | ***Model 2:***  **Financial help^b^**  *OR (95% CI)* | ***Model 3:***  **SNAP^c,d^**  *OR (95% CI)* | ***Model 4:***  **Food assistance^c,e^**  *OR (95% CI)* | ***Model 5:***  **Utility assistance^c,f^**  *OR (95% CI)* |
| No VD or dementia | *Reference* | *Reference* | *Reference* | *Reference* | *Reference* |
| VD Alone | 2.0 (1.3, 3.2) | 1.5 (1.0, 2.4) | 1.8 (1.2, 2.8) | 2.5 (1.4, 4.4) | 1.3 (0.8, 2.0) |
| Dementia Alone | 1.8 (1.1, 3.1) | 1.1 (0.8, 1.6) | 1.7 (1.1, 2.8) | 2.1 (1.1, 4.3) | 1.1 (0.6, 2.0) |
| Dementia & VI | 1.5 (0.6, 3.7) | 1.7 (1.1, 2.7) | 3.2 (1.7, 5.8) | 4.1 (2.0, 8.3) | 1.0 (0.5, 1.9) |
| *VD, Vision Difficulty; NHATS, National Health and Aging Trends Study; SNAP, Supplemental Nutrition Assistance Program*; *OR, odds ratio; CI, confidence interval*  ^a^ Only pay the minimum amount due in response to- “Do you usually pay off all credit card balances every month or only the minimum amount due?”  ^b^ “Last year, did you receive any financial help or gifts from relatives, either regularly -- like every month -- or just every so often as needed?”  ^c^ “There are several state and federal programs that help people in need. In the last year, did you receive help from any of these programs?”  ^d^ Food stamps (also called the Supplemental Nutrition Assistance Program, or SNAP)  ^e^ Other food assistance such as Meals-on-Wheels?  ^f^ Gas, electricity, or other energy assistance?  Models adjusted for NHATS participants’ age, sex, race/ethnicity, education, marital status, number of children, comorbidities, and hearing impairment. | | | | | |

| **Supplementary Table 2:** Multivariable Regression Analyses: Economic outcomes for caregivers of NHATS older adults by VD and probable/possible dementia status, accounting for clustering by care recipient, NSOC 2015 | |
| --- | --- |
| Group | ***Model 1:***  ***Financial Difficulty^a^***  *OR (95% CI)* |
| No VD or dementia | *Reference* |
| VD Alone | 1.3 (0.7, 2.4) |
| Dementia Alone | 1.4 (0.8, 2.4) |
| Dementia & VI | 3.0 (1.7, 5.4) |
| *VD, Vision Difficulty; NHATS, National Health and Aging Trends Study; NSOC, National Study of Caregiving; OR, odds ratio; CI, confidence interval*  ^a^ Caregivers were asked if helping the NHATS participant was financially difficult for them  Models adjusted for NHATS participants’ age, sex, race/ethnicity, education, marital status, number of children, comorbidities, and hearing impairment, and NSOC caregiver age, sex, education, self-reported health, and relationship to the older adult. | |
